# Prognostic role of nodal ratio, LODDS, pN in patients with pancreatic cancer with venous involvement

**DOI:** 10.1186/s12893-017-0311-1

**Published:** 2017-11-23

**Authors:** Giovanni Ramacciato, Giuseppe Nigri, Niccolo’ Petrucciani, Antonio Daniele Pinna, Matteo Ravaioli, Elio Jovine, Francesco Minni, Gian Luca Grazi, Piero Chirletti, Giuseppe Tisone, Fabio Ferla, Niccolo’ Napoli, Ugo Boggi

**Affiliations:** 1 Department of Medical and Surgical Sciences and Translational Medicine, Faculty of Medicine and Psychology, St Andrea Hospital, Sapienza University, General Surgery Unit, Via di Grottarossa 1037, 00189 Rome, Italy; 2 Department of Medical and Surgical Sciences-DIMEC, S. Orsola-Malpighi Hospital, Alma Mater Studiorum, University of Bologna, General Surgery and Transplantation Unit, Bologna, Italy; 3 General Surgery Unit, ‘Maggiore’ Hospital, Bologna, Italy; 4 Department of Medical and Surgical Sciences (DIMEC), Alma Mater Studiorum, S. Orsola-Malpighi Hospital, University of Bologna, General Surgery Unit, Bologna, Italy; 5 Regina Elena National Cancer Institute IFO, Hepato-pancreato-biliary Surgery Unit, Rome, Italy; 6 Department of Surgical Sciences, Sapienza University of Rome, Policlinico Umberto I Hospital, General Surgery Unit, Rome, Italy; 7 Department of Experimental Medicine and Surgery, Liver Unit, Tor Vergata University of Rome, Rome, Italy; 8 Division of General Surgery and Transplantation Surgery, Niguarda Hospital, Milan, Italy; 9 Division of General Surgery and Transplantation Surgery, Pisa University Hospital, Pisa, Italy

**Keywords:** Pancreatic cancer, Tnm, Nodal ratio, Lodds, Prognosis, Nodal staging, Venous invasion, Portal vein, Superior mesenteric vein, Pancreatectomy

## Abstract

**Background:**

The UICC/AJCC TNM staging system classifies lymph nodes as N0 and N1 in pancreatic cancer. Aim of the study is to determine whether the number of examine nodes, the nodal ratio (NR) and the logarithm odds of positive lymph nodes (LODDS) may better stratify the prognosis of patients undergoing pancreatectomy combined with venous resection for pancreatic cancer with venous involvement.

**Methods:**

A multicenter database of 303 patients undergoing pancreatectomy in 9 Italian referral centers was analyzed. The prognostic impact of number of retrieved and examined nodes, NR, LODDS was analyzed and compared with ROC curves analysis, Pearson test, univariate and multivariate analysis.

**Results:**

The number of metastatic nodes, pN, the NR and LODDS was significantly correlated with survival at multivariate analyses. The corresponding AUC for the number of metastatic nodes, pN, the NR and LODDS were 0.66, 0.69, 0.63 and 0.65, respectively. The Pearson test showed a significant correlation between the number of retrieved lymph nodes and number of metastatic nodes, pN and the NR. LODDS had the lower coefficient correlation. Concerning N1 patients, the NR, the LODDS and the number of metastatic nodes were able to significantly further stratify survival (*p* = 0.040; *p* = 0.046; *p* = 0.038, respectively).

**Conclusions:**

The number of examined lymph nodes, the NR and LODDS are useful for further prognostic stratification of N1 patients in the setting of pancreatectomy combined with PV/SMV resection. No superiority of one over the others methods was detected.

## Background

Pancreatic cancer represents the fourth-leading cause of cancer-related mortality in the United States with an estimated 53,670 new cases in 2017 and 43,090 deaths [1]. In Europe, an estimated 103,773 new cases were reported in 2012 [2]. Lymph nodal status is an important prognostic factor in these patients, as a determinant for the appropriate prognostic stratification and therapeutic decision-making [3]. Patients with pancreatic carcinoma with portal vein (PV) and/or superior mesenteric vein (SMV) invasion represent a particular challenge regarding prognostic analysis and treatment. The seventh edition of the International Union against Cancer (UICC) and the American Joint Committee on Cancer (AJCC) Tumor Node Metastasis (TNM) staging system classify regional lymph nodes as N0 and N1, according to the presence of none or one or more nodal metastases [4]. The number of lymph nodes should be reported because it represents a prognostic factor, and N0 patients have a better prognosis with an increasing number of examined lymph nodes [5–8]. For optimal staging, the analysis of 11–17 lymph nodes is recommended [5–9]. However, extended lymphadenectomy does not provide a survival advantage, according to randomized trials and meta-analyses [10–13]. In light of these data, the International Study Group of Pancreatic Surgery (ISGPS) agreed on a definition of standard lymphadenectomy [14]. Inaccurate surgical dissection, pathological evaluation or both may cause understaging for the suboptimal number of analyzed nodes, and subsequent inappropriate prognostic evaluation and error in clinical decisions [15].

To optimize nodal staging in patients with pancreatic cancer, different systems have been proposed and studied. The nodal ratio (NR) (ratio between metastatic and retrieved nodes) permits a subclassification of N1 patients, but it does not provide more information than TNM for N0 patients. Several authors have shown that the NR is a significant prognostic factor for overall survival [16, 17]. LODDS (log odds of positive lymph nodes), defined as the logarithm of the ratio between the number of positive nodes and number of negative nodes, has thus been proposed as more effective than the NR in N0 patients [15].

Until now, few studies have compared all nodal staging systems in patients with pancreatic carcinoma and no data have been published on patients with PV/SMV venous invasion. Therefore, our aim is to analyze and compare different nodal staging systems in a subgroup of patients who underwent pancreatectomy with combined venous resection in nine Italian referral centers in order to identify the more advantageous nodal classification in this subset of patients.

## Methods

The study included 303 patients who underwent pancreatectomy combined with PV or SMV resection for pancreatic carcinoma. The procedures were performed at nine Italian referral institutions. Some data retrieved from this multicenter database have already been published [18]. Written informed consent for participation in the study was obtained from participants.

### Preoperative work-up and surgical treatment

The diagnosis of pancreatic adenocarcinoma was confirmed by pathological examination in all cases. Regarding neoadjuvant treatment, the multidisciplinary board of each unit established the indication and protocol, after imaging discussion among radiologists and clinicians.

Pancreaticoduodenectomy, left spleno-pancreatectomy or total pancreatectomy were performed according to the site of the lesion. Lymphadenectomy was performed as previously described in a standard fashion [14].

### Definition of clinical outcomes and pathological examination

Postoperative mortality was defined as death during hospitalization or during the the first 30 days after pancreatectomy. For postoperative complications, ISGPS definitions were used [19, 20]. The presence of tumor cells within 1 mm from the margin was defined as R1 resection. Evidence of macroscopic residual tumor was defined as R2. ISGPS recommendations were followed [21].

### Lymph nodal staging systems

N status was defined according to the AJCC staging system. The nodal ratio (NR) was defined as the ratio between the number of positive nodes and total harvested nodes. The classification system validated by Malleo et al. was chose for NR, after a careful review of the literature. Interval values were as follows: 0, 0.01–0.2, 0.21–0.4 and more than 0.4 [3]. LODDS were calculated by log (pnod + 0.5)/(tnod-pnod + 0.5), where pnod was the number of positive lymph nodes and tnod was the total number of examined nodes; 0.5 was added to both the numerator and the denominator to avoid an infinite number [15]. Patients were divided into two groups, dichotomizing the LODDS values around the median value. Each subgroup was further divided into two, dichotomizing again around the median LODDS value, resulting in four LODDS groups. The classification reported by Strobel et al. was chosen for the number of positive nodes, [8].

### Adjuvant therapies and follow-up

The multidisciplinary tumor board of each institution validated the indication for adjuvant chemotherapy or radiochemotherapy. Decision was based on patients’ performance status and pathological results. During follow-up, physical examination and CA 19–9 determination were scheduled every 3 months in the first 2 years and than every 6 months, and thoraco-abdominal CT scan every 6 months in the first 2 years.

### Statistical analysis

The multicenter database was prospectively collected by each center and retrospectively analyzed. T-test for continuous variables and the chi-square test for categorical variables were used to calculate differences in distribution. Overall survival rates were calculated according to the Kaplan–Meier method, and we used the log-rank test to assess the statistical differences between different groups. Univariate and multivariate analyses were used to identify the most significant. Correlation between nodal staging systems and prognosis was assessed with univariate and multivariate analyses. Variables significant in the univariate analysis were used for the multivariate model, analyzing separately each nodal staging system. Overall survival rates were calculated according to different pN, NR, LODDS, number of metastatic nodes and number of retrieved nodes. ROC curves analysis was used to evaluate the accuracy of the different nodal staging systems, using nodal classifications as variables and 5-year survival as classification variables. The method of Delong et al. [22] was used. Pearson correlation coefficient was used to calculate the correlations between number of retrieved nodes, number of metastatic nodes, NR and LODDS. *p* < 0.05 was considered as statistically significant. Statistical analyses were performed by using MedCalc for Windows, version 10.2.0.0 (MedCalc Software, Belgium).

## Results

### Patients’ characteristics, preoperative work-up and treatment and surgery

The study population was composed of 165 men (54.5%) and 138 women (45.5%). The majority of patients were classed as N1 (70.6%). Patients’ characteristics according to nodal status are listed in Table 1. One hundred and eighty-seven patients (61.7%) had one or more comorbidity. Cardiovascular comorbidities were detected in 49.5% of patients, respiratory comorbidities in 12.2% and metabolic comorbidities in 29.0%.

**Table 1:** Patients’ characteristics and procedures. Data are presented for the entire cohort and according to nodal status. Continuous variables are presented as mean ± SD

Variable	N0 (89)	N1 (214)	Total (303)	*p*
Age	65.7 ± 10.7	67.4 ± 10.5	66.9 ± 10.6	0.223
Sex, Females	40.7%	47.5%	45.5%	0.348
ASA score	2.3 ± 0.7	2.5 ± 0.7	2.4 ± 0.7	0.379
BMI	23.8 ± 2.9	24.1 ± 3.2	24.0 ± 3.1	0.651
Comorbidities	57.0%	63.5%	61.7%	0.381
CEA (UI/ml)	15.7 ± 33.7	10.7 ± 19.1	12.0 ± 23.6	0.452
CA 19.9 (UI/ml)	592 ± 1276	644 ± 1375	637.0 ± 1345	0.831
Albumin (g/dl)	3.8 ± 0.6	3.7 ± 0.6	3.7 ± 0.6	0.258
Bilirubin (mg/dl)	6.7 ± 15.7	6.2 ± 6.3	6.3 ± 9.7	0.745
Tumor diameter at CT, mm	36.3 ± 22.6	31.6 ± 11.7	32.9 ± 15.4	0.103
Surgery				0.747
PD, number	68	165	233	
LP, number	18	42	60	
TP, number	3	7	10	

*SD* standard deviation, *ASA* American Society of Anesthesiology, *BMI* Body Mass Index, *CEA* carcinoembryonic antigen, *CA 19.9* Carbohydrate Antigen 19.9, *CT* computed tomography, *PD* pancreaticoduodenectomy, *LP* left pancreatectomy, *TP* total pancreatectomy

Mean tumor diameter according to the CT scan was 32.9 ± 15.4 mm. Preoperative biliary drainage was performed in 28.6% of cases, and neoadjuvant chemotherapy was administered to 6.4% of patients.

The majority of patients underwent pancreaticoduodenectomy (PD) (76.9%), 60 (19.8%) underwent left pancreatectomy (LP) and 10 (3.3%) total pancreatectomy (TP) (Table 1). All patients underwent portal or superior mesenteric vein resection. Mean operative time was 462.6 ± 134.2 min and blood loss 475.2 ± 401.6 ml.

### Postoperative outcomes, pathological and lymph nodal analysis and survival

Complications occurred in 49.8% of patients and mortality in 6.6% (Table 2). Postoperative pancreatic fistula occurred in 11.9% and delayed gastric emptying in 23.1%. Mean intensive care unit stay was 3.2 ± 4.6 days. Mean hospital stay was 20.4 ± 11.6 days. Histological venous invasion was found in 53.8% of venous specimens. Mean tumor diameter was 35.0 ± 20.7 mm. The mean number of retrieved lymph nodes was 33.5 ± 22.6 and ranged from 2 to 131. The mean number of metastatic nodes was 3.4 ± 4.5, ranging from 0 to 25. Patients undergoing LP had a significantly higher number of retrieved lymph nodes than patients undergoing PD (47.9 ± 25 versus 29.6 ± 20.2; *p* < 0.0001). The mean number of metastatic nodes was not different in patients submitted to PD (3.1 ± 4), LP (4.4 ± 5.8) and TP (3.7 ± 4.9). The resection margin was tumor-free in 73.3% of cases. Adjuvant therapy was administered to 72.1% of patients. Mean follow-up duration was 37.9 months. Median overall survival was 25 months and five-year survival rate was 25.2%.

**Table 2:** Postoperative complications and mortality in 303 patients submitted to pancreatectomy with portal vein and/or superior mesenteric vein resection

Variable	N.	%
Overall complications	151	49.8%
Mortality	20	6.6%
Pancreatic fistula	36	11.9%
Grade A	16	
Grade B	14	
Grade C	6	
DGE	70	23.1%
Grade A	32	
Grade B	25	
Grade C	13	
Non pancreatic leak	9	3.0%
Postoperative bleeding	18	5.9%
Re-laparotomy	21	6.9%
PV-SMV thrombosis	5	1.7%
Abdominal collection	33	10.9%
Need of postoperative abdominal drain	36	11.9%
Wound infection	16	5.3%
Urinary tract infection	3	1.0%
Cardiovascular complications	6	2.0%
DVT/PE	5	1.6%
Acute renal failure	3	1.0%
Pneumonia	4	1.3%

*N* number, *DGE* delayed gastric emptying, *PV-SMV* portal vein-superior mesenteric vein, *DVT/PE* deep venous thrombosis/pulmonary embolism

### Analysis of prognostic factors

**Fig. 1:**
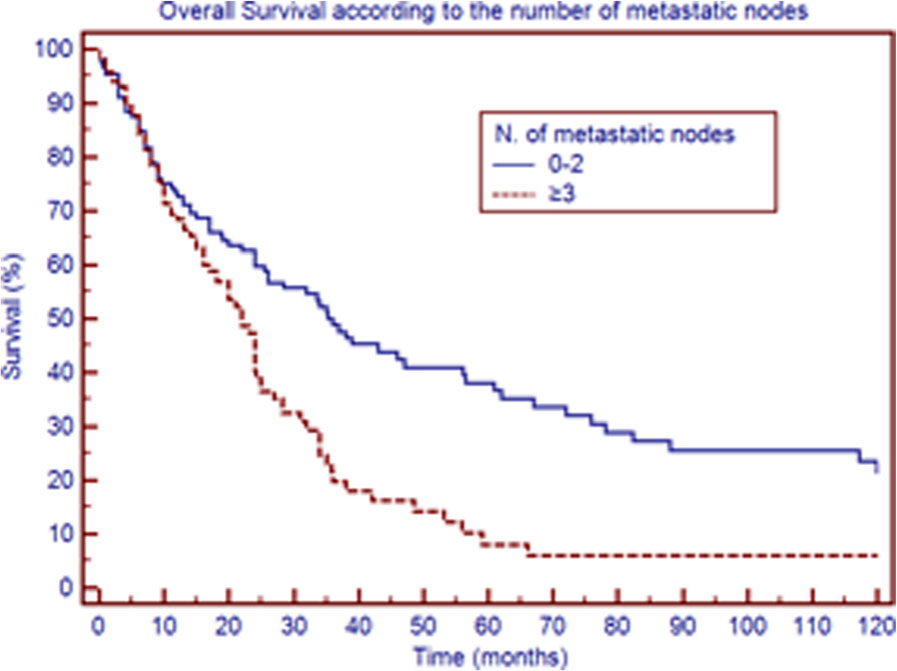
Overall survival according to the number of metastatic nodes

**Fig. 2:**
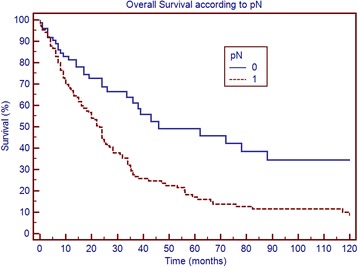
Overall survival according to pN status

**Fig. 3:**
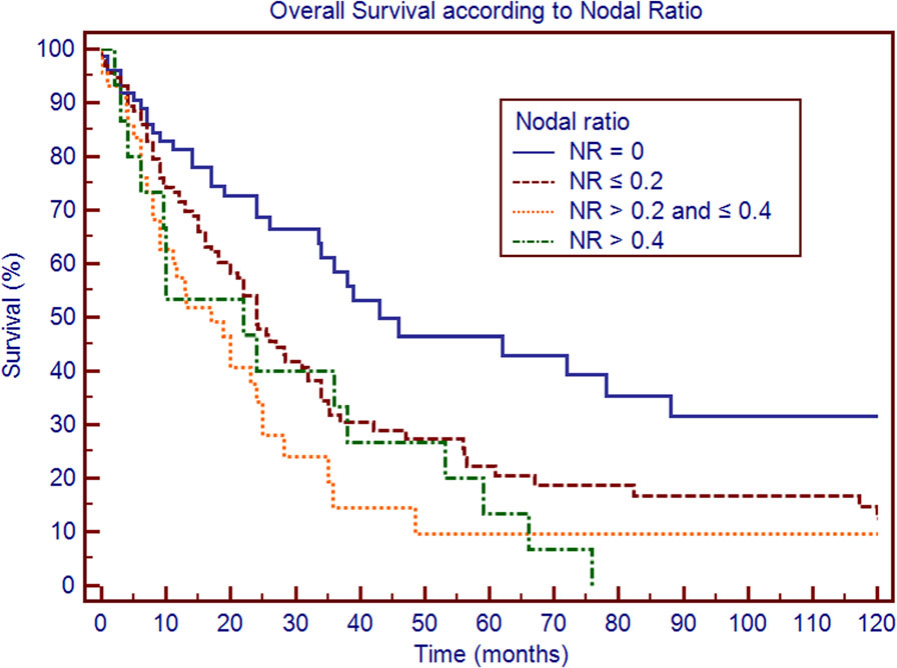
Overall survival according to the NR

**Fig. 4:**
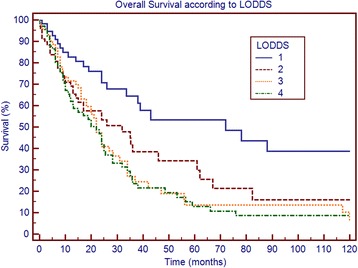
Overall survival according to LODDS

Table 3 shows survival according to patient and tumor characteristics. Factors that significantly correlated with overall survival were the number of metastatic nodes, pN, the NR and LODDS, whereas no correlation was found for age, sex, comorbidities, tumor size, number of retrieved nodes, T stage and resection margin. Figs. 1, 2, 3 and 4 show the survival curves according to the number of metastatic nodes, pN, the NR and LODDS, respectively. According to the multivariate analyses, the number of metastatic nodes, pN, the NR and LODDS were significantly correlated with survival (Table 4).

**Table 3:** Clinicopathological data and univariate survival analysis results of 303 patients submitted to pancreatectomy with portal vein and/or superior mesenteric vein resection

Variable	Patients (%)	Median survival (months)	*p* (univariate analysis)
Age			0.531
< 70	53.2	28.3	
*≥* 70	46.8	24	
Sex			0.094
Males	54.5	25	
Females	45.5	26	
Comorbidities			0.058
No	38.3	26	
Yes	61.7	24	
Tumor size			0.193
<30 mm	37.3	28	
*≥*30 mm	62.7	24	
Resection margin			0.850
R0	73.3	27	
R1	26.7	23	
T stage			0.506
1	0.7	na	
2	6.6	28	
3	86.1	24	
4	6.6	22	
Number of retrieved lymph nodes			0.797
< 17	24.1	26	
*≥* 17	75.9	24	
Number of metastatic lymph nodes			0.0005
0–2	56.1	35	
*≥* 3	43.9	22	
N stage	0.0002		
N0	29.4	46	
N1	70.6	23	
NR	0.0005		
0	29.4	43	
0.01–0.2	48.2	24	
0.21–0.4	16.2	17	
> 0.4	6.3	22	
LODDS			0.0013
Lodds <−0.005	23.4	72	
−0.005 ≤ Lodds <0.012	24.8	32	
0.012 ≤ Lodds <0.026	26.1	22	
Lodds ≥0.026	25.7	22	

*NR* nodal ratio, *LODDS* log odds of positive lymph nodes

**Table 4:** Multivariate analyses using the Cox proportional hazard method. Evaluation of prognostic impact of number of metastatic nodes, pN, nodal ratio and LODDS

Variable	b	SE	*P*	Exp (b)	95% CI
Age	−0.100	0.184	0.585	0.905	0.633 to 1.294
Sex	−0.244	0.188	0.196	0.784	0.543 to 1.132
Tumor size	0.176	0.197	0.372	1.192	0.813 to 1.749
R status	−0.089	0.221	0.686	0.915	0.594 to 1.407
T stage	−0.059	0.193	0.759	0.942	0.646 to 1.374
N. met. Nodes	0.427	0.186	0.022	1.533	1.066 to 2.23
Age	−0.013	0.183	0.945	0.987	0.691 to 1.411
Sex	−0.247	0.189	0.192	0.781	0.540 to 1.130
Tumor size	0.190	0.197	0.336	1.209	0.823 to 1.775
R status	−0.085	0.218	0.698	0.919	0.600 to 1.407
T stage	−0.115	0.195	0.555	0.892	0.610 to 1.303
N stage	0.603	0.239	0.011	1.828	1.148 to 2.911
Age	−0.0812	0.183	0.655	0.922	0.646 to 1.316
Sex	−0.195	0.190	0.305	0.823	0.568 to 1.192
Tumor size	0.206	0.196	0.293	1.229	0.838 to 1.803
R status	−0.170	0.221	0.441	0.843	0.548 to 1.298
T stage	−0.019	0.188	0.920	0.981	0.680 to 1.416
Nodal ratio	0.384	0.108	0.001	1.468	1.189 to 1.811
Age	−0.010	0.183	0.955	0.989	0.692 to 1.415
Sex	−0.229	0.188	0.221	0.795	0.551 to 1.146
Tumor size	0.184	0.197	0.349	1.202	0.819 to 1.764
R status	−0.189	0.222	0.395	0.828	0.537 to 1.277
T stage	−0.114	0.197	0.563	0.892	0.608 to 1.310
LODDS	0.273	0.089	0.002	1.313	1.105 to 1.562

*N. met. Nodes* number of metastatic nodes, *NR* nodal ratio, *LODDS* log odds of positive lymph nodes

### Comparison between pN staging, the NR and LODDS methods

**Fig. 5:**
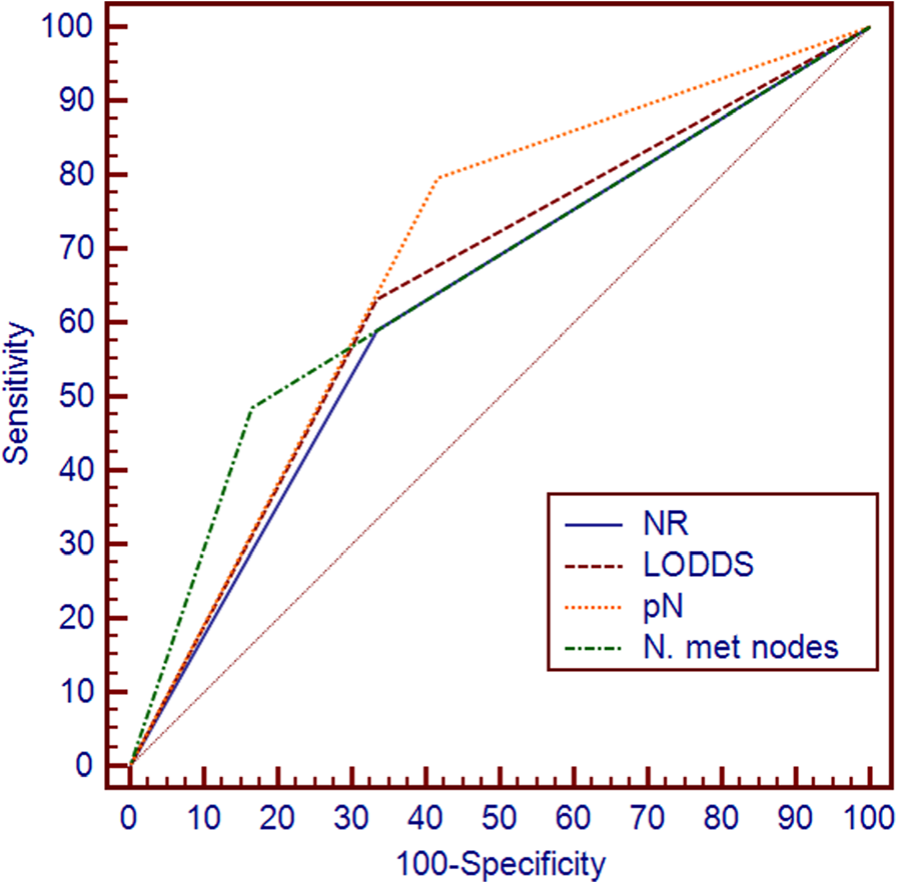
Comparison of ROC curves analysis between the number of metastatic nodes, pN status, the NR and LODDS

**Fig. 6:**
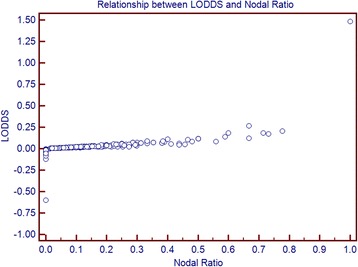
Relationship between LODDS and the NR

The corresponding AUC for the number of metastatic nodes, pN, the NR and LODDS were 0.66 (95% CI 0.58 to 0.73), 0.69 (95% CI 0.62 to 0.76), 0.63 (95% CI 0.55 to 0.70) and 0.65 (95% CI 0.57 to 0.72), respectively, with no significant differences (Fig. 5). The scatter plot of the relationship between LODDS and the NR is reported in Fig. 6. The LODDS value increased with the ratio of metastatic lymph nodes, showing correlation between LODDS and the NR. Values of LODDS were still heterogeneous, even in cases with NR = 0. A significant correlation between the number of retrieved lymph nodes and number of metastatic nodes was found at Pearson test (Table 5). The correlation values were lower for pN and the NR, and LODDS had the lower coefficient correlation (0.079) with the number of retrieved nodes (*p* = 0.154).

**Table 5:** Pearson correlation test between number of retrieved lymph nodes and number of metastatic nodes, N status, NR, LODDS

Correlation between of retrieved lymph nodes and number or metastatic nodes	
Sample size	303
Correlation coefficient r	0.298
Significance level	*p* < 0.0001
95% coefficient interval for r	0.192 to 0.398
Correlation between of retrieved lymph nodes and N status	
Sample size	303
Correlation coefficient r	0.1276
Significance level	*p* = 0.026
95% coefficient interval for r	0.015 to 0.237
Correlation between of retrieved lymph nodes and N ratio	
Sample size	303
Correlation coefficient r	−0.193
Significance level	*p* = 0.001
95% coefficient interval for r	−0.299 to −0.082
Correlation between of retrieved lymph nodes and LODDS	
Sample size	303
Correlation coefficient r	−0.082
Significance level	*p* = 0.154
95% coefficient interval for r	−0.193 to 0.031

*NR* nodal ratio, *LODDS* log odds of positive lymph nodes

### Further stratification of N1 and N0 patients

Concerning N1 patients, the NR was able to further stratify survival, with patients with NR < 0.2 having better prognosis (median survival of 24 versus 19 months, *p* = 0.040). Further, LODDS stratified N1 patients into two groups with different prognoses, with patients of LODDS >0.03 having significantly worse survival (20 versus 24 months, *p* = 0.046). In addition, the number of metastatic nodes was able to stratify N1 patients in this series, according to the classification reported by Strobel et al. (*p* = 0.038). In the group of patients with <17 retrieved lymph nodes, pN, the NR and LODDS were all able to stratify patient survival (*p* = 0.01, *p* = 0.023 and *p* = 0.05, respectively). The LODDS classification was used to stratify the 89 N0 patients; LODDS1 patients had a median survival of 72 months, whereas LODDS2 patients had a median survival of 36 months (not statistically significant; *p* = 0.229).

## Discussion

Lymph nodal status is considered to be one of the most important prognostic factors after pancreatectomy for adenocarcinoma. The most used nodal staging system is the N status of the AJCC classification, which identifies N0 and N1 patients, according to the presence or absence of nodal metastases. Previous studies have analyzed the prognostic role of the number of examined lymph nodes, number of pathologic lymph nodes, the NR and LODDS in patients with pancreatic cancer, with different results [5, 6, 8, 23, 24]. The number of positive nodes has been suggested to stratify N1 patients, adding prognostic information [8, 25]. Strobel and colleagues reported a median survival of 31.1, 26.1, 21.9 and 18.3 months in patients with 1, 2–3, 4–7 and >7 positive nodes, respectively [8]. The role of the number of positive nodes was also shown in patients submitted to pancreatic surgery after neoadjuvant therapy [25]. Concerning the NR, a number of authors have demonstrated its ability to further stratify node-positive patients [26, 27].

LODDS are new prognostic parameters, which aim to better stratify patients regarding their nodal metastases status. In the setting of gastric, colorectal, breast and other neoplasms, promising data have been reported [28–30]. Comparing to NR, which is a function of the number of retrieved nodes, LODDS is a function of the number of negative lymph nodes. In the setting of pancreatic cancer, only one study has analyzed this parameter, suggesting the advantage of LODDS over the NR in node-negative patients [15]. Patients with pancreatic cancer and portal vein/superior mesenteric vein axes involvement represent a peculiar and challenging subset of patients. Several questions are still open in this setting regarding better perioperative treatment, surgical strategies and prognostic stratification. No study has thus far analyzed the nodal staging system in this subset of patients to our knowledge, and for these reasons we reviewed a multicenter database to report our data about nodal prognostic factors in patients with venous invasion.

Our study analyzed a population of 303 patients undergoing pancreatectomy combined with venous resection. Patients were treated in referral centers for pancreatic pathology, and standard lymphadenectomy, as recommended by the ISGPS, was performed. The mean number of retrieved lymph nodes was high (33.5), and the majority of patients (70.6%) had at least one metastatic node. Patients submitted to LP had a higher number of retrieved nodes, whereas the number of metastatic nodes was not different in patients undergoing PD, LP or TP. Univariate and multivariate analyses of prognostic factors were performed. Nodal staging indexes were significant predictors of survival, and the multivariate analysis confirmed the significant prognostic value of the number of metastatic nodes, pN, the NR, and LODDS. A comparison of the different systems was attempted to demonstrate the superiority of one of them. The ROC curves’ comparison did not show any significant differences. LODDS had a lower and non-significant correlation with the number of retrieved nodes according to the Pearson test, which may be advantageous in the case of inadequate lymphadenectomy (in this series, only 12.5% of patients has fewer than 11 retrieved nodes). A scatter plot was presented to show that LODDS has the power to discriminate patients with the same NR (0 or 1) but a different prognosis. However, in the entire cohort, all nodal staging systems seemed to be efficacious with a strong prognostic significance.

We further studied the group of patients having at least one nodal metastasis. Clearly, pN classification is limited in this setting, because all patients are classified as N1. The comparison of survival curves via the log-rank test demonstrated that the NR, the number of positive nodes and LODDS might all provide further stratification for these patients. This result is concordant with those of previous studies, and confirms that pN staging may also be integrated by further information. The number of positive nodes is easy to retrieve and does not require calculation. However, patients with different prognosis may have the same values. For example, the number of positive nodes is the same for patients having 4 metastatic nodes out of 4 retrieved (100% of metastatic nodes) or 4 out of 40 (10%), for example. The NR is simple to calculate. NR carries information that are related to both the number of metastatic and retrieved nodes. However, for values approaching 1 its accuracy seems to diminish (no difference between a patient with 1/1 metastasis and one with 40/40). Furthermore, further stratification of N0 patients is not possible using NR and number of positive nodes. LODDS represent a nodal prognostic index, which is more complex to understand. Furthermore, calculation is less simple, which explain why is rarely used in clinical practice. Theoretically, LODDS have several advantages, including the possibility to further stratify N0 patients. In our series, we failed to demonstrate a statistically significant difference in the 89 N0 patients using LODDS, but these results were limited by the sample size of N0 patients in our study population.

The novelty of this study is that it is the first one evaluating different nodal staging systems in the setting of patients undergoing pancreatectomy with synchronous venous resection. Patients with portal vein or superior mesenteric vein invasion represent a challenging subset of patients, and optimal prognostic stratification is needed in their clinical management. We demonstrated that N1 patients might be further classified using the number of examined lymph nodes, the NR and LODDS. Furthermore, our study adds useful information on the role of LODDS and pancreatic cancer, which is still controversial. Only a few studies have been published about LODDS in pancreatic cancer staging, with some authors suggesting its utility [15] and others recommending avoiding its use [31].

We point out some limitations of this study. Data regarding disease-free survival were not analyzed, because not all included centres reported the information. Furthermore, the study is retrospective. However, use of ISGPS definition and the numbers of included patients represent some remarkable aspects of this series. In this study, neoadjuvant therapy was administered only to a minority of patients. We can explain this data analyzing NCCN guidelines until 2014. Up-front surgery was indicated in fit patients with venous invasion at CT scan suitable to resection and reconstruction with complete tumor clearance. Furthermore, neoadjuvant therapy is a factor that may modify nodal status; hence, the low rate of neoadjuvant therapy in this study represents an advantage regarding the analysis of nodal prognostic factors.

## Conclusions

In conclusion, in patients undergoing pancreatectomy with combined PV/SMV resection for pancreatic cancer, the number of examined lymph nodes, the NR and LODDS are useful for the further prognostic stratification of N1 patients. All these staging systems permit the better stratification of patients with nodal metastases and are useful in clinical practice. No superiority of one over the others was detected in patients undergoing pancreatectomy with venous resection.

## References

[1] Siegel RL, Miller KD, Jemal A (2017). Cancer statistics, 2017. CA Cancer J Clin.

[2] New European Cancer Observatory – IARC; Cancer factsheets. Available at: http://eco.iarc.fr/eucan/Cancer.aspx?Cancer=15. Accessed 13 Jul 2015.

[3] Malleo G, Maggino L, Capelli P (2015). Reappraisal of nodal staging and study of lymph node station involvement in pancreaticoduodenectomy with the standard international study group of pancreatic surgery definition of lymphadenectomy for cancer. J Am Coll Surg.

[4] Pancreas Cancer Staging, American Joint Committee on Cancer, 7^th^ Edition. Available at: https://cancerstaging.org/references-tools/quickreferences/Documents/PancreasSmall.pdf. Accessed 1 Apr 2016.

[5] Valsangkar NP, Bush DM, Michaelson JS (2013). N0/N1, PNL, or LNR? The effect of lymph node number on accurate survival prediction in pancreatic ductal adenocarcinoma. J Gastrointest Surg.

[6] Huebner M, Kendrick M, Reid-Lombardo KM (2012). Number of lymph nodes evaluated: prognostic value in pancreatic adenocarcinoma. J Gastrointest Surg.

[7] Opfermann KJ, Wahlquist AE, Garrett-Mayer E, Shridhar R, Cannick L, Marshall DT (2014). Adjuvant radiotherapy and lymph node status for pancreatic cancer: results of a study from the surveillance, epidemiology, and end results (SEER) registry data. Am J Clin Oncol.

[8] Strobel O, Hinz U, Gluth A (2015). Pancreatic adenocarcinoma: number of positive nodes allows to distinguish several N categories. Ann Surg.

[9] Ashfaq A, Pockaj BA, Gray RJ, Halfdanarson TR, Wasif N (2014). Nodal counts and lymph node ratio impact survival after distal pancreatectomy for pancreatic adenocarcinoma. J Gastrointest Surg.

[10] Michalski CW, Kleeff J, Wente MN, Diener MK, Büchler MW, Friess H (2007). Systematic review and meta-analysis of standard and extended lymphadenectomy in pancreaticoduodenectomy for pancreatic cancer. Br J Surg.

[11] Sun J, Yang Y, Wang X (2014). Meta-analysis of the efficacies of extended and standard pancreatoduodenectomy for ductal adenocarcinoma of the head of the pancreas. World J Surg.

[12] Jang JY, Kang MJ, Heo JS (2014). A prospective randomized controlled study comparing outcomes of standard resection and extended resection, including dissection of the nerve plexus and various lymph nodes, in patients with pancreatic head cancer. Ann Surg.

[13] Dasari BV, Pasquali S, Vohra RS (2015). Extended versus standard lymphadenectomy for pancreatic head cancer: meta-analysis of randomized controlled trials. J Gastrointest Surg.

[14] Tol JA, Gouma DJ, Bassi C (2014). Definition of a standard lymphadenectomy in surgery for pancreatic ductal adenocarcinoma: a consensus statement by the international study group on pancreatic surgery (ISGPS). Surgery.

[15] La Torre M, Nigri G, Petrucciani N (2014). Prognostic assessment of different lymph node staging methods for pancreatic cancer with R0 resection: pN staging, lymph node ratio, log odds of positive lymph nodes. Pancreatology.

[16] Liu ZQ, Xiao ZW, Luo GP (2014). Effect of the number of positive lymph nodes and lymph node ratio on prognosis of patients after resection of pancreatic adenocarcinoma. Hepatobiliary Pancreat Dis Int.

[17] Slidell MB, Chang DC, Cameron JL (2008). Impact of total lymph node count and lymph node ratio on staging and survival after pancreatectomy for pancreatic adenocarcinoma: a large, population-based analysis. Ann Surg Oncol.

[18] Ramacciato G, Nigri G, Petrucciani N, et al. Pancreatectomy with mesenteric and portal vein resection for borderline resectable pancreatic cancer: multicenter study of 406 patients. Ann Surg Oncol. 2016; [Epub ahead of print] 10.1245/s10434-016-5123-5 26893222

[19] Bassi C, Dervenis C, Butturini G (2005). Postoperative pancreatic fistula: an international study group (ISGPF) definition. Surgery.

[20] Wente MN, Bassi C, Dervenis C (2007). Delayed gastric emptying (DGE) after pancreatic surgery: a suggested definition by the international study Group of Pancreatic Surgery (ISGPS). Surgery.

[21] Bockhorn M, Uzunoglu FG, Adham M (2014). Borderline resectable pancreatic cancer: a consensus statement by the international study Group of Pancreatic Surgery (ISGPS). Surgery.

[22] DeLong ER, DeLong DM, Clarke-Pearson DL (1988). Comparing the areas under two or more correlated receiver operating characteristic curves: a nonparametric approach. Biometrics.

[23] Kang MJ, Jang JY, Chang YR, Kwon W, Jung W, Kim SW (2014). Revisiting the concept of lymph node metastases of pancreatic head cancer: number of metastatic lymph nodes and lymph node ratio according to N stage. Ann Surg Oncol.

[24] Riediger H, Keck T, Wellner U (2009). The lymph node ratio is the strongest prognostic factor after resection of pancreatic cancer. J Gastrointest Surg.

[25] Fischer LK, Katz MH, Lee SM (2016). The number and ratio of positive lymph nodes affect pancreatic cancer patient survival after neoadjuvant therapy and pancreaticoduodenectomy. Histopathology.

[26] Yamada S, Fujii T, Hirakawa A, Kanda M, Sugimoto H, Kodera Y. Lymph node ratio as parameter of regional lymph node involvement in pancreatic cancer. Langenbeck's Arch Surg. 2016; [Epub ahead of print] 10.1007/s00423-016-1412-5 27048402

[27] Robinson SM, Rahman A, Haugk B (2012). Metastatic lymph node ratio as an important prognostic factor in pancreatic ductal adenocarcinoma. Eur J Surg Oncol.

[28] Wen J, Ye F, He X, et al. Development and validation of a prognostic nomogram based on the log odds of positive lymph nodes (LODDS) for breast cancer. Oncotarget. 2016; 10.18632/oncotarget.8091 PMC4991511 26992235

[29] Song YX, Gao P, Wang ZN (2011). Which is the most suitable classification for colorectal cancer, log odds, the number or the ratio of positive lymph nodes?. PLoS One.

[30] Aurello P, Petrucciani N, Nigri GR (2014). Log odds of positive lymph nodes (LODDS): what are their role in the prognostic assessment of gastric adenocarcinoma?. J Gastrointest Surg.

[31] Riediger H, Kulemann B, Wittel U (2016). Prognostic role of log odds of lymph nodes after resection of pancreatic head cancer. J Gastrointest Surg.

